# Broncho-Vaxom Attenuates Allergic Airway Inflammation by Restoring GSK3β-Related T Regulatory Cell Insufficiency

**DOI:** 10.1371/journal.pone.0092912

**Published:** 2014-03-25

**Authors:** Ran Fu, Jian Li, Hua Zhong, Dehong Yu, Xianping Zeng, Mengxia Deng, Yueqi Sun, Weiping Wen, Huabin Li

**Affiliations:** 1 Allergy and Cancer Center, Otorhinolarygology Hospital, The First Affiliated Hospital of Sun Yat-sen University, Guangzhou, China; 2 Department of Otolaryngology, Head and Neck Surgery, Xinhua Hospital, Shanghai Jiaotong University School of Medicine, Shanghai, China; University of KwaZulu-Natal, South Africa

## Abstract

**Background:**

Oral administration of bacterial extracts (eg, Broncho-Vaxom (BV)) has been proposed to attenuate asthma through modulating Treg cells. However, the underlying mechanism has not been fully characterized. This study sought to assess the effects of oral administration of BV on GSK-3β expression and Treg cells in ovalbumin (OVA)-induced asthmatic mice models.

**Method:**

Asthmatic mice models were established with OVA challenge and treated with oral administration of BV. Next, infiltration of inflammatory cells including eosinophil and neutrophils, mucous metaplasia, levels of Th1/Th2/Treg-typed cytokines and expression of GSK3β and Foxp3 were examined in asthmatic mice models by histological analysis, Bio-Plex and western blot, respectively. Moreover, the frequencies of Treg cells were evaluated in cultured splenocytes by flow cytometry in the presence of BV or GSK3β siRNA interference.

**Results:**

We found significant decrease of infiltrated inflammatory cells in bronchoalveolar lavage fluid (BALF) in asthmatic mice models after oral administration of BV. Oral administration of BV was shown to significantly suppress mucus metaplasia, Th2-typed cytokine levels and GSK3β expression while increasing Foxp3 production in asthmatic mice models. Moreover, BV significantly enhanced GSK3β-related expansion of Treg cells in cultured spleen cells *in vitro*.

**Conclusion:**

Our findings provide evidence that oral administration of BV is capable of attenuating airway inflammation in asthmatic mice models, which may be associated with GSK3β-related expansion of Treg cells.

## Introduction

Allergic airway inflammation such as allergic rhinitis and asthma is characterized by skewed Th2 response, allergen-specific IgE-mediated eosinophilic inflammation, mucus hypersecretion and airway hyperresponsiveness [1]. Some studies suggest the insufficient suppression of Treg cells, which may account for the excessive Th2 response in asthma, plays a critical role in initiating allergic sensitization and driving airway hyperresponsiveness [2], [3]. On the other hand, it has been proposed that baseline airway Treg activity in sensitized rats can be boosted by microbe-derived stimulation of the gut, resulting in enhanced suppressive capacity to control asthma [4]. Therefore, inducing Treg cells through oral administration of bacterial extracts to attenuate the established Th2 response may represent a promising treatment option for allergic rhinitis and asthma.

The pathophysiology of allergic inflammation has not been fully understood [5]. As suggested by the hygiene hypothesis, the increased allergy prevalence is probably as a result of changes in environmental factors including reduced exposure to microbial antigens [6]. Accordingly, early exposure to pathogens and infection with various parasites was associated with prevention of asthma [7]. The inverse correlation between exposure to food-borne microbes and respiratory allergy has therefore pointed toward a role for the gut and gut-associated lymphoid tissue in shaping the immune response against environmental antigens [8]. Currently, probiotics treatment through the gastrointestinal mucosa has been shown to attenuate systemic as well as local immunoinflammatory functions [9]. This protective effect is thought to be associated with the regulation of common mucosal immune system including lymphoid cells that “programmed” in the gut and subsequently home to other mucosal sites to modulate local inflammatory response.

Broncho-Vaxom (BV) is an endotoxin-low, lyophilized fractionated alkaline extract of the following eight bacterial strains: Haemophilus influenzae, Diplococcus pneumonia, Klebsiella pneumoniae, Klebsiella ozaenae, Staphylococcus aureus, Streptococcus pyogenes, Streptococcus viridans, and Neisseria catarrhalis. It has been widely used in children and adults suffering from repeated upper respiratory tract infections, and it efficiently reduces both the frequency and the duration of the infections [10]. Some studies have recently shown that BV can induce Treg in vitro and in vivo that confers protection against asthma [11], [12]. However, the mechanisms by which Treg responses are stimulated by bacterial extracts remains unclear. GSK3β is a serine/threonine protein kinase involved in glycogen metabolism and the Wnt signalling pathway, which plays important roles in embryonic development and tumourigenesis [13]. GSK3β is known to regulate the activity of NF-κB, which has been associated with the development of asthma [14]. Accordingly, inhibitor of GSK-3β significantly inhibited ovalbumin (OVA)-induced airway inflammation and reduced airway hyperresponsiveness to inhaled methacholine in asthmatic mice models [15]. Recently, it has been shown that inhibition of GSK-3β resulted in increased suppression activity by Treg cells [16], suggesting GSK3β-mediated Treg insufficiency may be considered a therapeutic target for the management of asthma.

## Materials and Methods

### Ethics Statement

The research protocols were approved by the Ethics Committee of the First Affiliated Hospital of Sun Yat-sen University.

### Establishment and Pretreatment of Asthmatic Mice Model

**Figure 1:**
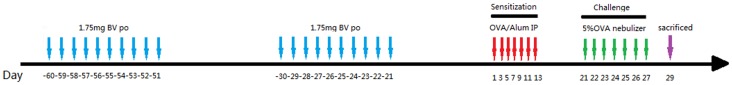
Experimental protocol for different groups of asthmatic mice models. The OVA-sensitized and challenged group: mice were sensitized on days 1, 3, 5, 7, 9, 11 and 13 by i.p. injection of 40 μg OVA plus 5 mg Al(OH)_3_. One week later, mice were challenged with 5% OVA aerosol inhalation (Compressing Nebulizer, Yuyue, China) for 30 minutes on 7 consecutive days (between day 21 to day 27). PBS was used for oral administration before OVA sensitization and challenge.

Male BALB/c mice aged 6–8 weeks were obtained from the Guangdong Medical Experimental Animal Centre (Guangzhou, China) and housed under pathogen-free conditions. The experiment protocol was shown in Fig 1, and asthmatic mice models were established as we described elsewhere [17]. Briefly, mice were sensitized on days 1, 3, 5, 7, 9, 11 and 13 by intraperitoneal (i.p.) injection of 40 μg OVA plus 5 mg Al(OH)_3_. One week later, mice were challenged with 5% OVA aerosol inhalation (Compressing Nebulizer, Yuyue, China) for 30 minutes on 7 consecutive days (between day 21 to day 27). For pretreatment of bacterial extract, 1.75 mg commercially available Broncho-Vaxom (BV) (OM-PHARMA, Meyrin/Geneva, Switzerland) were intragastric (i.g.) administrated for 10 consecutive days and rested for 20 days as a circle. 36 BALB/c mice were randomly divided into 6 groups (n = 6 per group) (oral administration/sensitization/challenge) as follows: (1) PBS/OVA/OVA group: mice were sensitized and challenge by OVA, PBS was used for oral administration before OVA sensitization and challenge; (2) 1 mon BV/OVA/OVA pretreated group: mice were orally administrated with BV for 1 circle before OVA sensitization and challenge; (3) 2 mon BV/OVA/OVA pretreated group: mice were orally administrated with BV for 2 circles before OVA sensitization and challenge; (4) 1 mon BV/PBS/PBS pretreated group: mice were orally administrated with BV for 1 circle before PBS sensitization and challenge; (5) 2 mon BV/PBS/PBS pretreated group: mice were orally administrated with BV for 2 circles before PBS sensitization and challenge; (6) PBS/PBS/PBS group: mice were orally administrated with PBS for 2 circles, PBS was used for sensitization and challenge.

All mice were sacrificed via cervical dislocation under anesthesia 2 days after stimulation. The level of serum OVA-specific IgE was examined using commercial ELISA kits (Shibayagi, Shibukawa, Gunma, Japan) following the manufacturer's instructions. Pulmonary tissues were examined using haematoxylin-eosin (HE), periodic acid-Schiff (PAS) and immunohistochemical (IHC) staining.

### Number of Inflammatory Cells in Bronchoalveolar Lavage Fluid (BALF) in Asthmatic Mice Models

The nasal secretions from nasopharynx to the nostril and the lung, were perfused with 0.4 mL ×3 times PBS containing 1% fetal bovine serum after partial tracheal resection using 22-gauge catheters, and then BALF was collected as we described elsewhere [17]. After centrifugation, the inflammatory cells in BALF were counted using a hemocytometer and then cytospun onto glass slides and stained with Diff-Quick (Baso Diagnostics Inc., Zhuhai, China). A total of 300 cells per slide were evaluated for eosinophils, macrophages, neutrophils, and lymphocytes in 5 high-power fields (HPFs, magnification, 400×) under light microscope and averaged.

### Bio-Plex Cytokine Assay

The levels of IL-1β, IL-2, IL-4, IL-5, IL-6, IL-10, IFN-β and TGF-β1 in BALF of asthmatic mice models, as well as the levels of IL-4, IL-10, TGF-β1 and IFN-γ in the supernatants of cultured splenocytes, were measured by using the Bio-Plex mouse Cytokine Panel assay (Bio-Rad, Hercules, CA, USA) according to the manufacturer's instruction.

### Histological Examination of Pulmonary Tissues in Asthmatic Mice Model

Pulmonary tissues were examined using HE, PAS and IHC staining. For evaluation of the goblet cell hyperplasia, the percentage of PAS-positive cells in epithelial areas was counted in 5 HPFs (magnification, 400×) and averaged. Every pulmonary tissue was examined for 3 different sections. Moreover, the total number of eosinophils in the pulmonary tissues was similarly counted in 5 HPFs (magnification, 400×). The lung inflammation score was performed in a reproducible scoring system. Briefly, five sections across the main bronchus of each animal were randomly selected and given scores ranging from 0 to 3 based on the level of peribronchial and perivascular inflammation to quantify the lung inflammation. The values were given according to the following inflammatory parameters: 0, no inflammation was detectable; 1, occasional cuffing with inflammatory cells; 2, most bronchi or vessels surrounded by a thin layer (1–5 cells) of inflammatory cells; and 3, most bronchi or vessels were surrounded by a thick layer (more than five cells) of inflammatory cells.

For IHC staining, 4-μm-thick paraffin-embedded sections were deparaffinised, rehydrated, and washed with PBS. The sections were incubated with 0.01 mol/L sodium citrate (pH6.0) in 95°C water for 20 minutes and 0.5% triton X-100 for 10 minutes, blocked with 10% goat serum (Invitrogen, Carlsbad, CA, USA) for 30 minutes and then incubated with anti-human GSK3β antibody (1∶200) (Cell Signaling, Danvers, MA, USA) overnight at 4°C. Thereafter, each section was incubated with a secondary antibody and then with horseradish peroxidase-labeled streptavidin complex (Zhongshanjinqiao, Beijing, China). Distribution of peroxidase was revealed by incubating the sections in a solution containing 3% 3,3-diaminobenzidine tetrahydrochloride before being counterstained with hematoxylin and coverslipped. Negative control studies were performed by replacing the primary antibodies with normal IgG in appropriate concentrations. The integrated optical density in each section was calculated in 5 HPFs (magnification, 400×) using Image-Pro Plus 6.0 software (Media Cybernetics Inc, MD, USA) and averaged.

### Real-time Quantitative PCR

Total RNA was isolated from tissue samples with Trizol (Invitrogen) according to the manufacturer's instructions. RNA was reverse transcribed by ReverTra Ace qPCR RT Kit (Toyobo Biochemicals, Osaka, Japan) according to the manufacturer's protocol. Sequence-specific primers for Foxp3, GSK3β and GAPDH were as follows: Foxp3 forward, 5′- CAC CTA TGC CAC CCT TAT CCG -3′, Foxp3 reverse, 5′-CAT GCG AGT AAA CCA ATG GTA GA -3′, GSK3β forward, 5′- CAG TGG TGT GGA TCA GTT GG-3′, GSK3β reverse, 5′- CAA TTG CCT CTG GTG GAG TT -3′, GAPDH forward, 5′-AGG TCG GTG TGA ACG GAT TTG-3′, GAPDH reverse, 5′-TGT AGA CCA TGT AGT TGA GGT CA-3′. Real-time PCR was performed with GoTaq qPCR Master Mix (Promega, Madison, WI, USA) on a MiniOpticon Real-time PCR detection instrument (Bio-Rad) using the SyBr Green detection protocol as outlined by the manufacturer. Briefly, the amplification mixture consisted of 0.5 μM primers, 25 μl of GoTaq qPCR Master Mix, and 1 μl template DNA in a total volume of 40 μl. Samples were amplified with the following program: initial denaturation at 98°C for 30 sec, followed by 40 cycles of denaturation for 15 s at 98°C and annealing/elongation for 60 s at 60°C. All PCRs were run in triplicate, and control reactions without template were included. The fluorescent signals were collected during the extension phase, Ct values of the sample were calculated, target gene mRNA levels were normalized by GAPDH and compared with PBS/PBS/PBS group, and analyzed by 2^−ΔΔCt^ method.

### Western Blot Analysis

The protein expression of GSK3β in the pulmonary tissues of asthmatic mice models and stimulated spleen cells was examined by western blot analysis. Total protein was extracted from pulmonary tissues or cultured spleen cells in of RIPA lysis buffer at 4°C for 30 min. The protein concentration was determined by the Bradford method. Samples containing 10 μg of protein were boiled, subjected to SDS-PAGE in 10% Tris-glycine gels and transferred electrophoretically to polyvinylidene fluoride membranes. The membranes were incubated with 5% fat-free skim milk in Tris-buffered solution (TBS) 81 containing 0.05% Tween 20 (1 h, room temperature) and then incubated with anti-human GSK3β antibody (1∶1000) (Cell Signaling) overnight at 4°C. The membrane was then incubated with horseradish peroxidase-linked secondary antibody and finally processed using the ECL chemiluminescence reaction kit (Cell Signalling), followed by exposure on medical film. The relative band density of phosphorylated protein was quantified with the Bio-Rad Quantity One 1-D Analysis Software (Bio-Rad).

### Statistical Analysis

The data were expressed as the mean ± SD. Statistical analysis was performed with one-way analysis of variance (ANOVA) followed with Tukey post test, Student's *t*-test for comparison between groups, Student-Newman-Keuls for Homogeneous Subsets analyses. *P*-values<0.05 were considered to be significant.

## Results

### Oral Administration of BV Modulated Inflammatory Cells and Cytokine Levels in BALF of Asthmatic Mice Models

**Figure 2:**
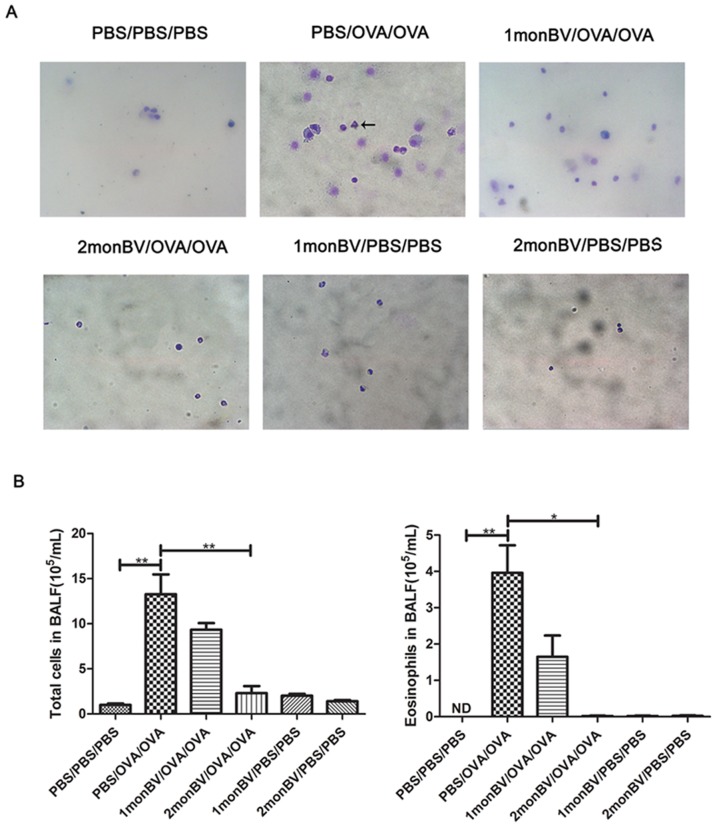
Oral administration of BV decreased infiltrated inflammatory cells in BALF of asthmatic mice models. (A), Representative results of infiltrated inflammatory cells in BALF of asthmatic mice models, as suggested by Diff-quick staining. (B), The infiltrated eosinophils, neutrophils and other inflammatory cells were significantly decreased in BALF of asthmatic mice models in 2 monBV/OVA/OVA group. The total inflammation cells and eosinophils in each sample were counted at 5 high power fields (HPFs) and averaged (magnification ×400) (n = 6). ND, no detection. Each sample was tested for 3 times and averaged, all values were expressed as mean ± SD. * *P*<0.05.

**Figure 3:**
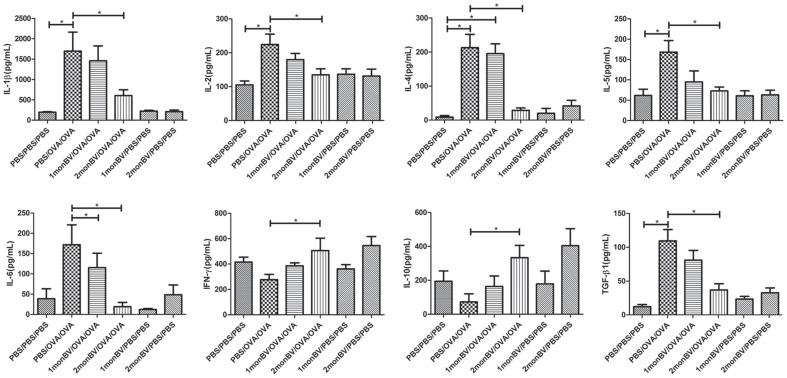
Oral administration of BV modulated cytokine levels in BALF of asthmatic mice models. The levels of IL-1β, IL-2, IL-4, IL-5, IL-6, IL-10, IFN-γ and TGF-β1 in BALF of asthmatic mice models were examined by Bio-Plex. Oral administration of BV significantly decreased the levels of IL-1β, IL-4, IL-5 and TGF-β1 but increased the levels of IFN-γ and IL-10 in BALF of asthmatic mice models in 2 monBV/OVA/OVA group (n = 6). Each sample was tested for 3 times and averaged, all values were expressed as mean ± SD. * *P*<0.05.

To evaluate the pretreated effects of oral BV administration on established allergic response, we firstly examined the serum level of OVA-specific IgE, and found the level of OVA-specific IgE in 2 mon BV/OVA/OVA pretreated group was significantly decreased when compared to that in PBS/OVA/OVA group (data not shown). Next, we examined the infiltrated inflammatory cells in BALF of asthmatic mice models. As shown in Fig 2, we found the infiltrated eosinophils, neutrophils and other inflammatory cells were significantly increased in BALF of asthmatic mice models, which were significantly inhibited in asthmatic mice models in 2 mon BV/OVA/OVA pretreated group (*P*<0.05). Accordingly, the levels of IL-1β, IL-2, IL-4, IL-5, IL-6, IL-10, IFN-γ and TGF-β1 in BALF of asthmatic mice models were differentially modulated by oral BV administration as well. As shown in Fig 3, the levels of IL-1β, IL-4, IL-5 and TGF-β1 were significantly increased in asthmatic mice models after OVA challenge, which were significantly inhibited in asthmatic mice models in 2 mon BV/OVA/OVA pretreated group (*P*<0.05). However, oral BV administration significantly increased the levels of IFN-γ and IL-10 in BALF of asthmatic mice models in 2 mon BV/OVA/OVA pretreated group, which were significantly decreased in established asthmatic mice models (*P*<0.05).

### Oral Administration of BV Modulated Airway Inflammatory Cells and Mucous Metaplasia in Asthmatic Mice Models

**Figure 4:**
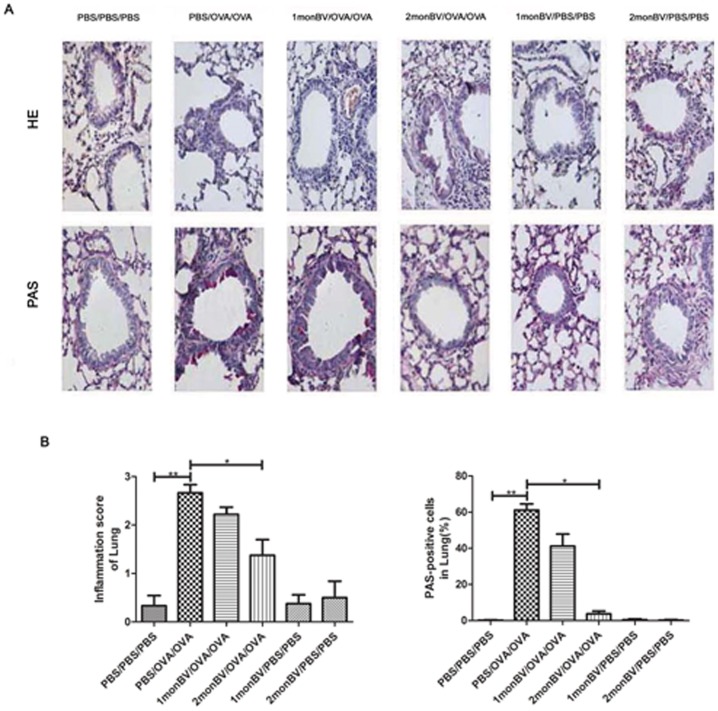
Oral administration of BV attenuated allergic airway inflammation and mucous metaplasia in asthmatic mice models. (A), Representative histological results of pulmonary tissues in asthmatic mice models, as suggested by HE and PAS stainings (magnification ×400). (B), The toal number of eosinophils and mean number of PAS-positive cells in the pulmonary tissues in 2 monBV/OVA/OVA group, as counted at 5 high power fields (magnification ×400) (n = 6). Each sample was tested for 3 times and averaged, all values were expressed as mean ± SD. **P<0.01. * *P*<0.05.

To evaluate the effects of oral BV administration on allergic pulmonary inflammation, we examined the histological changes in pulmonary tissues of asthmatic mice models using HE and PAS staining. As shown in Fig 4, OVA challenge induced marked infiltration of inflammatory cells into the pulmonary tissues and enhanced mucous metaplasia as compared with PBS challenge, the majority of the infiltrated inflammatory cells were eosinophils. The mean number of eosinophils (as suggested by HE staining) and PAS-positive cells (as suggested by PAS staining) were significantly increased in pulmonary tissues in asthmatic mice models, which were significantly attenuated in 2 mon BV/OVA/OVA pretreated group (*P*<0.05).

### Oral Administration of BV Modulated Expression of GSK3β and Foxp3 in Asthmatic Mice Models

**Figure 5:**
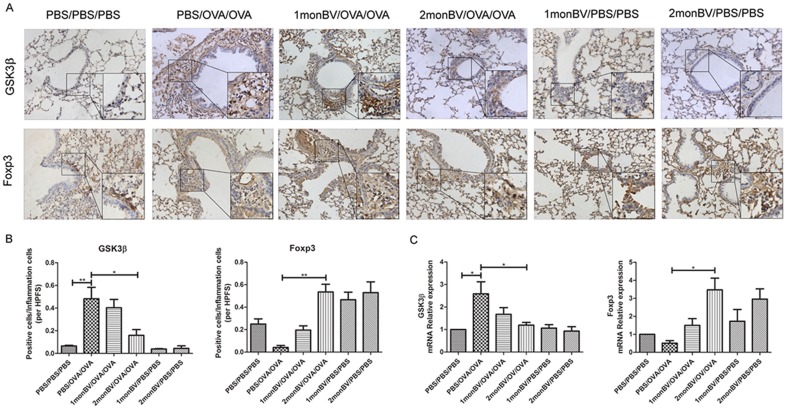
Oral administration of BV modulated GSK3β and Foxp3 expression in the pulmonary tissues of asthmatic mice models. (A), Representative immunohistochemical results of GSK3β and Foxp3 expression in the pulmonary tissues of asthmatic mice models (magnification ×400). (B), Oral administration of BV significantly decreased the ratio of GSK3β^+^ cells, but increased the ratio of Foxp3^+^ cells, in the pulmonary tissues of asthmatic mice models in 2 monBV/OVA/OVA group, as indicated by integrated optical density (n = 6). (C), The mRNA expression levels of GSK3β and Foxp3 were showed by RT-QPCR, when normalized by GAPDH and compared with PBS/PBS/PBS group. Each sample was tested for 3 times and averaged, all values were expressed as mean ± SD. * *P*<0.05.

Moreover, as shown in Fig 5, we found OVA challenge significantly induced GSK3β expression as compared with PBS challenge. The immunoreactivity of GSK3β was extensively distributed in the pulmonary tissues. When evaluated the effect of oral BV administration on GSK3β expression, we found the ratio of GSK3β^+^ cells was significantly inhibited in 2 mon BV/OVA/OVA pretreated group but not in 1 mon BV/OVA/OVA pretreated group (*P*<0.05). Conversely, the ratio of Foxp3^+^ cells, which was sparsely distributed in the pulmonary mucosa, was significantly decreased in pulmonary tissues in asthmatic mice models after OVA challenge (*P*<0.05). When evaluated the effect of oral BV administration on Foxp3 expression, we found the ratio of Foxp3^+^ cells was significantly increased in 2 mon BV/OVA/OVA pretreated group but not in 1 mon BV/OVA/OVA pretreated group (*P*<0.05). Interestingly, the expression of Foxp3 was also found to be upregulated in 1 mon and 2 mon BV/PBS/PBS pretreated group (*P*<0.05). Similarly, the mRNA expression level of GSK3β was increased in asthmatic mice model and decreased in 2 mon BV/OVA/OVA pretreated group (*P*<0.05). Conversely, the mRNA expression level of Foxp3 was decreased in asthmatic mice model and evaluated in 2 mon BV/OVA/OVA pretreated group (*P*<0.05).

### BV Modulates GSK3β-mediated Suppression of Foxp3^+^ Treg Cells *in vitro*

**Figure 6:**
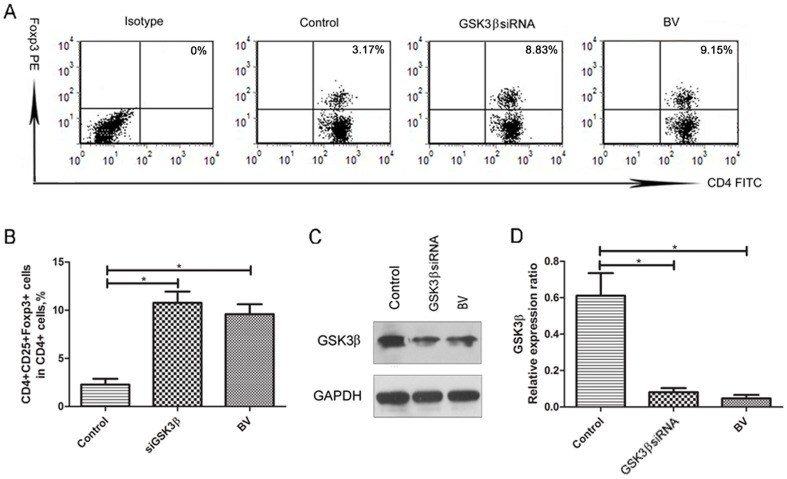
BV modulates GSK3β-mediated suppression of Foxp3^+^ Treg cells *in vitro*. (A), Representative flow cytometric results of Foxp^3+^ Treg cells in cultured splenocytes after incubation with BV or transfection with GSK3β siRNA for 72 h. (B), Both BV (100 μg/mL) and GSK3β siRNA (100 pmol/mL) were shown to significantly increase the frequencies of Foxp3+ Treg cells in cultured splenocytes. (C), Representative western blot results of GSK3β expression in cultured splenocytes after incubation with BV or transfection with GSK3β siRNA for 72 h. (D), Both BV and GSK3β siRNA were shown to significantly decrease the expression of GSK3β in cultured splenocytes (n = 3). Each sample was tested for 3 times and averaged, all values were expressed as mean ± SD. * P<0.05.

**Figure 7:**
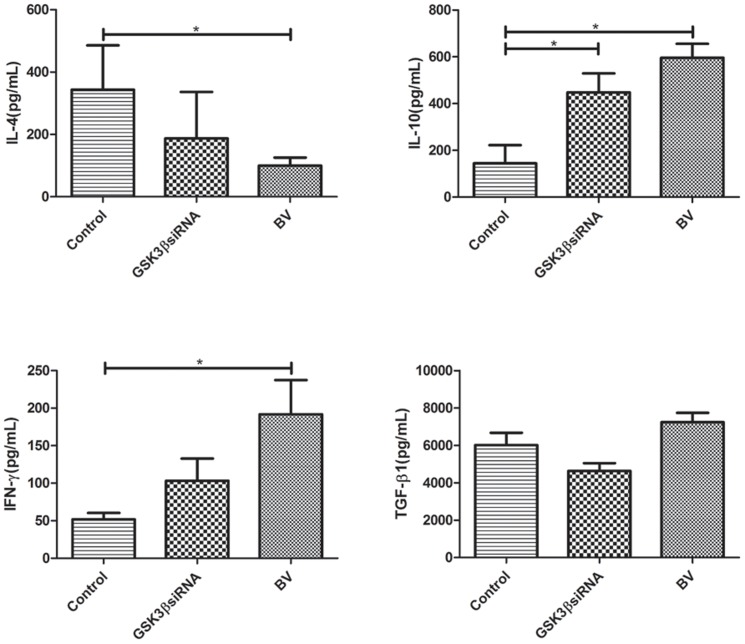
BV and GSK3β siRNA modulate the levels of cytokines in the supernatants of cultured splenocytes. Both BV (100 μg/mL) and GSK3β siRNA (100 pmol/mL) were shown to significantly decrease the levels of IL-4, but increase the levels of IL-10 and TGF-β1 in the supernatants of cultured splenocytes. The levels of cytokines were determined by Bio-Plex (n = 3). Each sample was tested for 3 times and averaged, all values were expressed as mean ± SD. * *P*<0.05.

To further characterize the possible pharmaceutical mechanism underlying BV administration on allergic response in asthmatic mice models, we then examined Foxp^3+^ Treg cells in cultured splenocytes in the *in vitro* assay (see Methods S1). Consequently, we found BV significantly increase the frequencies of Foxp^3+^ Treg cells in cultured splenocytes (P<0.05). To verify whether GSK3β was involved in BV-induced Foxp^3+^ Treg expansion *in vitro*, we examined the effect of GSK3β siRNA on the frequencies of Foxp^3+^ Treg cells in cultured splenocytes, and found GSK3β siRNA alone significantly increase the frequencies of Foxp^3+^ Treg cells in cultured splenocytes as well (P<0.05) (Fig 6A and B). As a correspondence, both BV and GSK3β siRNA were shown to significantly decrease the expression of GSK3β in cultured splenocytes, as suggested by western blot analysis (P<0.05) (Fig 6 C and D). Additionally, both BV and GSK3β siRNA were shown to significantly decrease the levels of IL-4, but increase the levels of IL-10 and TGF-β1 in the supernatants of cultured splenocytes (P<0.05) (Fig 7).

## Discussion

Gastrointestinal stimulation with microbe-derived agents represents a promising strategy for asthma control [4]. In the present study, we provided the first evidence that the expression of GSK3β and Foxp3 exerted a negative relationship in asthmatic mice models. Moreover, we showed that oral administration of BV was able to attenuate allergic airway response by modulating GSK3β-related Foxp3 expression and expansion of Treg cells. These findings therefore expanded our understanding on the pathophysiology of asthma and contributed to the establishment of optimal management of allergic airway diseases.

GSK3β is a serine-threonine kinase that is constitutively active in cells and has recently been demonstrated to play a critical role in the pathophysiology of asthma by regulating NF-κB activation [14]. In this study, we established a direct relationship between pro-inflammatory GSK3β and anti-inflammatory Foxp3 in asthmatic mice models. Up to now, the interplay between GSK3β and Foxp3 has not been fully clarified. Previously, Graham et al. reported that inhibition of GSK3β led to increased suppression activity by Treg cells, and GSK3β inhibitor-treated Treg cells exhibited prolonged Foxp3 expression and increased levels of β-catenin and of the antiapoptotic protein Bcl-xL [16]. These novel findings suggest that GSK3β may be a useful target in developing strategies designed to increase the stability and function of Treg cells for treating allergic diseases. Therefore, it is of interest to investigate whether oral administration of microbe-derived agents exerts anti-allergic property through modulating GSK3β/Treg axis.

Currently, the pathophysiology of allergic diseases has not been fully understood. Some epidemiological data points to the intestinal microbiota as playing a definitive role in influencing the immunological events that could lead to the development of allergic disorders [18]–[20]. Based on these findings, gastrointestinal stimulation with microbe-derived agents including probiotics and bacterial extracts has been suggested as a strategy for asthma control. For example, Strickland et al showed OM-85BV pretreatment significantly reduced allergic inflammation in BALF [4]. In Navarro et al's study [12], they showed oral administration of BV efficiently protects mice from asthma. Similarly, in Ahrens et al.' study [21], they reported no difference between OVA-sensitized animals treated or not treated with bacterial lysates, but showed oral administration of bacterial lysates attenuates experimental food allergy after challenge. It is important to note that there existed some difference in the experiment protocol of oral BV administration, and the clinical protocol of BV for respiratory infection was suggested as oral administration for 10 days and rest for 20 days as 1 circle. In the present study, we strictly followed the clinical protocol by orally administrated BV for 1 and 2 circles (oral administration for 10 days and rest for 20 days as 1 circle). In agreement with the previous studies, we found the serum level of OVA -specific IgE, infiltrated inflammatory cells and Th2 cytokine levels in BALF, as well as mucous metaplasia, were significantly inhibited in 2 mon but not 1 mon BV/OVA/OVA pretreated group. These findings suggested that only BV pretreatment for 2 circles is able to attenuate allergic response in asthmatic mice models. In regards to the time course that caused different effects, we supposed that BV needed more than 1 month to activate the secondary immune system which may lead to stronger immunomodulatory effect.

The molecular mechanism underlying BV-induced resolution of allergic airway response has not been fully understood. It has recently been proposed by Morandi et al. that bacterial lysates are able to induce activation of dendritic cells [22]. BV is an endotoxin-low, lyophilized fractionated alkaline extract of the following eight bacterial strains contained various pathogen associated molecular pattern, which may arise adaptive immune response by binding to pattern-associated receptor such as toll like receptors in dendritic cells, which can subsequently modulated T cell phenotypes [23]. In Parola et al.'s study [24], they reported that BV activates human dendritic cells via the NF-kB and MAPK pathways, which results in a mild pro-inflammatory activation of dendritic cells and favor the recruitment of innate effector cells to promote lymphocyte function.

Since Treg cells have a role in protecting against human allergic disease, numerous studies focused on the induction of Treg cells in BV-treated asthmatic mice models. For example, Strickland et al' showed the effects of OM-85BV treatment appear to be directed toward regulation of the CD86 response of dendritic cells, as well as induction of selective recruitment of Treg to airways [4]. In Navarro et al's study, they proposed oral treatment with BV suppressed airway inflammation through IL-10-dependent and MyD88-dependent mechanisms and induced the conversion of Foxp3^−^ T cells into Foxp3^+^ Treg cells [12]. However, the mechanisms by which Treg responses are stimulated by pathogens remains unclear. In this study, we provided the clue that BV's anti-allergic property in asthmatic mice models may be associated with modulation of GSK3β/Treg axis *in vivo* and *in vitro*. We acknowledged that this study may contain some flaws since it failed to show how GSK3β mediated BV's regulatory effect on Treg cells. Thus, further studies are still required to address what pathogen sensing receptors BV activated and what the downstream signaling pathways are involved in the regulation of Treg cells to improve the clinical efficacy for allergic airway diseases.

## Conclusion

In conclusion, our findings provided the evidence that BV exerts anti-allergic property in asthmatic mice models through modulating GSK3β/Treg axis. This newly identified mechanism will be beneficial to designing optimal strategy for the prevention of asthma and other allergic diseases.

## Supporting Information

Methods S1 *In vitro* Splenocyte Culture and Stimulation and Flow Cytometric Analysis of Foxp3^+^ Treg Cells.
(DOC)
