# Supplementary material for: Broncho-Vaxom Attenuates Allergic Airway Inflammation by Restoring GSK3β-Related T Regulatory Cell Insufficiency
Source: PLoS One. 2014 Mar 25;9(3):e92912. doi: 10.1371/journal.pone.0092912 (PMC3965496; doi:10.1371/journal.pone.0092912)
Supplement: Methods S1 — In vitro Splenocyte Culture and Stimulation and Flow Cytometric Analysis of Foxp3+ Treg Cells. (DOC) [file pone.0092912.s001.doc]

**Supporting Information**

**Materials and Methods**

*In vitro* Splenocyte Culture and Stimulation

Mice were intraperitoneal sensitized by OVA for 2 times (on day 1 and 7), then the spleen was harvested after 7 days’ rest. Single cell suspension of splenocyte was obtained by pressing spleens through a 40-μm cell strainer (BD Falcon, Bedford, MA, USA). Erythrocytes were removed using red blood cell lysing buffer (Sigma). Single splenocytes were cultured in complete medium containing RPMI 1640 supplemented with 10% heat-inactivated FBS, 2 mM l-glutamine, 50μm β-mercaptoethanol, 100 U/mL penicillin/100 μg/mL streptomycin (Invitrogen), 10ng/mL IL-2 (PeproTech, Rocky Hill, NJ, USA), 5μg/mL CD3 and 1μg/mL CD28 (R&D systems, Minneapolis, MO, USA) in 37°C, 5% CO_2_ incubator. Then BV (100 μg/mL) was added for incubation with cultured splenocytes for 48-72h. For siRNA transfection, specific GSK3β siRNA (sense sequence: CUG CCA UCG AGA CAU UAA ATT; antisense sequence: UUU AAU GUC UCG AUG GCA GTT) or mock siRNA was used. A mixture of siRNA and transfection reagent RNAiMAX (Invitrogen) in culture medium was incubated with 5× 10^6^ cells for 48-72h. Then cells and supernatants were separately collected for further use.

Flow Cytometric Analysis of Foxp3^+^ Treg Cells

For flow cytometric analysis, collected spleen cells (1× 10^6^) were stained with the CD4CD25Foxp3 phonotype testing kit (eBioscience, San Diego, CA, USA) according to the manufacturer’ instruction. Briefly, after washed twice with staining buffer, cells were incubated with anti-CD4-FITC and anti-CD25-APC for 45 minutes on ice, then washed 3 times with staining buffer and incubated with the fixation/ permeabilization buffer for 1 hour on ice in the dark. After washed with permeabilization buffer for 3 times, cells were incubated with anti-Foxp3-PE for 30 minutes on ice in the dark, and resuspended in staining buffer for analysis on a BD FACS Calibur Flow cytometer (BD Biosciences, San Jose, CA, USA). PE- and FITC-conjugated Rat IgG2a and APC-conjugated rat IgG1 antibodies were used as isotyped control. Results were analyzed using FCS Express V3 (De Novo Software, Canada). Each experiment was repeated for 3 times and averaged.
